# Notes on Trachelorrhaphy

**Published:** 1884-12

**Authors:** 


### Selections.

### Notes on Trachelorrhaphy.

Dr. Thad. A. Reamy, of Cincinnati, Chairman of the Obstetric Section of the American Medical Association, chose for the subject of his address before the general session, at the Washington meeting in May last, "Notes of Two Hundred and Thirty-one Cases of Operation for Laceration of the Cervix Uteri."

His first operation was made February 28, 1874. Not a single death has occurred in his 231 different patients operated upon. In six cases the operation was followed by perimetritis, parametritis, or peritonitis. These complications delayed, but did not prevent, ultimate recovery. In one case these inflammatory complications were attributed to the undue degree to which the uterus was dragged down, in order to give unobstructed access.

Of these 231 cases, in 170 the laceration was bilateral, in 38 unilateral. Of these latter, 23 were on the left side and 15 on the right. Sixteen cases were stellate, in two of which there were four distinct rents. In five cases there was laceration of the posterior lip only, in two of the anterior lip only. In 80 cases the laceration was extensive, in 15 of these 80 extending to the cervico-vaginal junction on both sides. In 22 of the bilateral cases the laceration extended to the cervico-vaginal junction on one side only. In three cases the rent extended to the internal os. In one of these the vaginal wall was extensively torn; the peritoneal cavity had probably been opened, followed by protracted cellulitis, and 12 sutures were required on one side to close the cervical and vaginal rent.

In 167 cases there was perineal laceration to an extent that left deformity, either at the vaginal orifice or more externally. In 15 cases the anal sphincter was damaged. In seven cases the recto-vaginal septum was opened up. In 26 cases the cervix and perineum were operated on at the same sitting. In five cases the uterus was curetted with the blunt wire at the same sitting, this being the primary of the three procedures. In 50 cases the perineum was operated upon after recovery from the cervical operation.

Dr. Reamy does not hesitate to curette the uterus in a case demanding it, at the time of operating upon the cervix, or the perineum, experience having convinced him that neither of these operations adds materially to the danger of curetting; but, on the other hand, he inclines to the opinion that in certain cases where the conjoined conditions demanding the two operations exist, the depletion of the cervix in trachelorrhaphy presents an element of safety against the inflammatory processes which may follow curetting. Secondary hemorrhage did not follow trachelorrhaphy in any of his cases. In every case where hemorrhage was troublesome during the operation, it was readily controlled by the stream of hot water which it is his custom to have flow upon the field of operation during its progress, with but three exceptions; in which it being necessary to denude deeply, twigs of the circular branch of the uterine artery being cut, compression with hemostatic forceps was invoked.

Dr. R. believes in the importance of allowing free bleeding from the denuded surfaces, firstly (*a*) because it softens the tissues so as to allow more perfect coaptation of the lips to be united; and (*b*) because of this softening, which is immediate, the tissues are more easily penetrated by the needles carrying the sutures. Secondly, and more important, this depletion not only greatly promotes the return of the tissue to its normal condition, but is an immense factor in again starting the processes of involution which had been arrested by the laceration and its consequences. Thorough denudation, cutting out all cicatricial tissue, and allowing free depletion, embrace the essential points in the operation so far as its influence upon involution is concerned.

Dr. R. believes that the early rupture of the membranes, attempts at forcible dilatation of the cervix by the fingers of the accoucheur or midwife, and the improper use of ergot, are more productive of lacerations of the cervix than the forceps. In Cincinnati 70 per centum of all labors are attended by midwives, who are generally ignorant and unskilful, and he thinks the sins above lie closely at their doors. These errors are, however, sometimes perpetrated by well-educated physicians, though many cases of laceration are, of course, unavoidable, even under the most skilful care. He counsels caution, therefore, against the indiscriminate charge of unskilfulness on the part of the accoucheur, lest some be unjustly censured.

Dr. Reamy takes position alongside most American gynecologists, in the view that the fretting of the exposed tissue consequent upon an ununited laceration of the cervix, is a prolific source of danger in developing cancer, and cites eminent authority, at home and abroad, in support thereof.

In regard to the necessity of the operation, he declares that it is an error to limit trachelorrhaphy to cases where ectropion has occurred, or where a cicatricial plug imprisons or presses upon branches of sentient nerves, causing painful reflex symptoms. He asserts that any laceration which has healed without its surfaces being in contact must have healed without complete union, although its extent may have been much lessened by granulation. In all such cases there is more or less cicatricial tissue in the field of repair, which, in such locality, even should it not produce reflex symptoms, should be thoroughly removed and the parts closed so as to obtain union by the first intention, in which cicatricial tissue is never found. If the rent be small, then the operation is small, especially if it be done before chronic inflammation of the cervix has occurred, as a result.

As bearing upon the question of its influence upon sterility, Dr. R. says he knows of 15 of his cases where conception has occurred with delivery at term, six of which he attended himself. In no case did re-lacerations occur, and the labors were normal.

In but two cases did union fail. This was due in one case to the use of the catgut ligature, and in the others to the sutures not being sufficiently tightened. In each case subsequent operation was successful.

He summarizes his method of operating as follows:

1. I use nothing to draw the uterus down with but the single volsella, with which I seize but one lip, the one to be denuded first, at a time.

2. I draw the uterus down as little as possible. This caution should be the more scrupulously observed, if any cellulitis remains about the base of the broad ligaments or elsewhere.

3. I outline the denudation with a sharp knife, and then cut the tissue included in the line with a sharp scissors. This prevents the rolling of the tissue at the borders.

4. Allow bleeding freely or not, as the condition of the tissue of the cervix, and the involution or subinvolution of the uterus, may require.

5. Use a nearly half-circle needle with very sharp point, armed with Chinese silk. This shaped needle can be drawn through the second lip and withdrawn very much more easily than a straight needle, or one curved only near the point.

Silk is preferred to wire, because it can be tied more quickly, and the tension more easily adjusted. Then there are no ends to jag the vaginal walls.

6. Wash out the cervical canal at the close of the operation with a recurrent flow syringe to remove any blood that may have found its way there during closure of the sutures. Have nurse wash out the vagina with warm, carbolized water within an hour after the operation is completed. The vagina is not syringed again until the sixth day, then daily until the patient is dismissed.

Cleanliness is the only antisepsis employed.—*Journal of the American Medical Association, Sept. 20, 1884.*

